# Dual Antiplatelet Therapy with Parenteral P2Y_12_ Inhibitors: Rationale, Evidence, and Future Directions

**DOI:** 10.3390/jcdd10040163

**Published:** 2023-04-09

**Authors:** Giulia Alagna, Paolo Mazzone, Marco Contarini, Giuseppe Andò

**Affiliations:** 1 Department of Clinical and Experimental Medicine, University of Messina, 98124 Messina, Italy; giulia.alagna@icloud.com; 2 Cardiology Unit, “Umberto I” Hospital, 96100 Siracusa, Italy; mazzonepaolo89@gmail.com (P.M.); m.contarini@asp.sr.it (M.C.)

**Keywords:** dual antiplatelet therapy, P2Y_12_ inhibitors, acute coronary syndrome, clopidogrel, prasugrel, ticagrelor, cangrelor, selatogrel, zalunfiban

## Abstract

Dual antiplatelet therapy (DAPT), consisting of the combination of aspirin and an inhibitor of the platelet P2Y_12_ receptor for ADP, remains among the most investigated treatments in cardiovascular medicine. While a substantial amount of research initially stemmed from the observations of late and very late stent thrombosis events in the first-generation drug-eluting stent (DES) era, DAPT has been recently transitioning from a purely stent-related to a more systemic secondary prevention strategy. Oral and parenteral platelet P2Y_12_ inhibitors are currently available for clinical use. The latter have been shown to be extremely suitable in drug-naïve patients with acute coronary syndrome (ACS), mainly because oral P2Y_12_ inhibitors are associated with delayed efficacy in patients with STEMI and because pre-treatment with P2Y_12_ inhibitors is discouraged in NSTE-ACS, and in patients with recent DES implantation and in need of urgent cardiac and non-cardiac surgery. More definitive evidence is needed, however, about optimal switching strategies between parenteral and oral P2Y_12_ inhibitors and about newer potent subcutaneous agents that are being developed for the pre-hospital setting.

## 1. Introduction

Dual antiplatelet therapy (DAPT) consists of the combination of aspirin and an inhibitor of the platelet P2Y_12_ receptor for adenosine diphosphate (ADP). At the end of the 1990s, two randomized trials definitively established DAPT with aspirin and ticlopidine as the gold standard therapy after percutaneous coronary intervention (PCI) with stent implantation, in comparison to aspirin or to aspirin and anticoagulant therapy [1,2]. Ticlopidine was soon replaced by clopidogrel at the beginning of the 2000s. DAPT has proven to be among the most investigated treatments in cardiovascular medicine. Such necessity of research initially arose from the observations of late and very late stent thrombosis (ST) events occurring after first-generation drug-eluting stent (DES) implantation, highlighting lack of efficacy of clopidogrel as one of the possible drivers of thrombotic events [3] and paving the way to development of potent oral agents such as prasugrel [4] and ticagrelor [5]. More recent evidence in high-risk patients has suggested that DAPT reduces the long-term risk of cardiovascular death, spontaneous myocardial infarction (MI), stroke and major adverse cardiac events (MACE) [6,7]. After decades of research, DAPT has been moving from a stent-related to a systemic treatment among other secondary prevention strategies such as lipid-lowering therapy and control of diabetes and hypertension. Most evidence remains largely based on post-PCI patients [8], while patients that are either medically managed (e.g., those with MINOCA [9], spontaneous coronary artery dissection [10], or takotsubo syndrome [11]) or undergoing coronary artery bypass grafting (CABG) [12] remain underrepresented in clinical trials. On this background, we will discuss the role, indications, and utilization of cangrelor, the only parenteral P2Y_12_ inhibitor available so far, its recommendations as a bridging antiplatelet agent for cardiac and non-cardiac surgery and the future directions of DAPT with new parenteral agents.

## 2. P2Y_12_ Inhibitor Antiplatelet Agents

### 2.1. Oral P2Y_12_ Inhibitors

While ticlopidine was the first P2Y_12_ inhibitor to be associated with low-dose aspirin for DAPT, its unfavorable safety profile made it obsolete after the introduction of clopidogrel. Clopidogrel is a second-generation thienopyridine and an irreversible P2Y_12_ receptor antagonist that is administered as an inactive pro-drug and requires enzymatic liver conversion into its active metabolite by a series of cytochrome P450 (CYP) enzymes. After activation, clopidogrel irreversibly binds to P2Y_12_, an ADP receptor, on the surface of platelets, resulting in an inactivation of the glycoprotein (GP) IIb/IIIa receptor and destabilization of the platelet aggregate [6]. The recommended regimen is a loading dose of 600 mg followed by a maintenance dose of 75 mg once daily. No dose adjustment is required in CKD patients. The onset of action is particularly delayed and variable, ranging from 2 to 6 h and the offset of effect ranges from 3 to 10 days. The evidence provided by the landmark CURE trial established DAPT with clopidogrel as the standard of care after acute coronary syndrome (ACS) and after coronary stent implantation [13]. However, clopidogrel has too much inter-individual variability in platelet inhibition and has significant non responsiveness and resistance in some patients. The enzymatic liver conversion is one of the main causes of variability of clopidogrel action. CYP2C19 is one of the most important polymorphic CYP enzymes across different populations and this is associated with worse outcomes, for instance, in those with the CYP2C19*2 variant [14]. Likewise, all comedications that are inhibitors of CYP2C19 suppress clopidogrel bioactivation (e.g., some proton pump inhibitors, statins and calcium channel blockers) [15]. Moreover, poor intestinal absorption can delay the onset of action of clopidogrel, which can be worsened by concomitant administration of opioids for angina relief. Inadequate P2Y_12_ inhibition, especially in the setting of ACS, contributes to more frequent periprocedural complications such as need for recurrent revascularization, MI, and ST. This highlighted the need for a more potent and consistent platelet inhibition that was introduced with novel generation P2Y_12_ inhibitors. 

Prasugrel is thienopyridine as well and an irreversible P2Y_12_ receptor antagonist that is administered as an inactive pro-drug and requires an enzymatic liver activation. Differently than clopidogrel, it gains a faster, greater, and more consistent degree of platelet inhibition [16]. The recommended regimen is a loading dose of 60 mg followed by a maintenance dose of 10 mg once daily, reduced to 5 mg in patients ≥75 years old or <60 kg. No dose adjustment is required in CKD patients. The onset of action is rapid, ranging from 0.5 to 4 h and the offset of effect ranges from 5 to 10 days. The TRITON-TIMI 38 trial compared prasugrel versus clopidogrel in P2Y_12_ inhibitor-naïve ACS patients referred to PCI [4]. Prasugrel determined a reduction in primary ischemic endpoint compared to clopidogrel, counterbalanced by a significant increase in the rate of major bleeding. Prasugrel was also compared to ticagrelor, the other potent P2Y_12_ inhibitor, in the recent ISAR-REACT 5 randomized trial. Prasugrel was superior in reducing the rate of death, MI, and stroke without any increase in bleeding complications [17]. Thus, prasugrel is the recommended P2Y_12_ inhibitor in ACS patients without high bleeding risk proceeding to PCI [18].

Ticagrelor is a direct oral reversible P2Y_12_ receptor inhibitor, which belongs to a novel chemical class, the cyclopentyl triazolopyrimidine. Following intestinal absorption, ticagrelor does not need to be metabolized for platelet inhibition. The recommended dose is a loading dose of 180 mg followed by a maintenance dose of 90 mg twice a day. No dose adjustment is required in CKD patients. The onset of action is rapid as well, ranging from 0.5 to 2 h and the offset of effect ranges from 3 to 4 days. The PLATO trial proved the superiority of ticagrelor compared to clopidogrel in ACS patients regarding the rate of death from vascular causes, MI, or stroke, without significant difference in major bleeding rates [5]. Nevertheless, ticagrelor also led to more patients stopping medication because of side effects, mainly dyspnea. As it is not associated with pulmonary or cardiac dysfunction, alterations in the mechanisms and the neurological pathways of the sensation of dyspnea may be involved in its pathogenesis [19].

### 2.2. Drawbacks of Oral P2Y_12_ Inhibitors

Despite potent P2Y_12_ inhibitors (prasugrel and ticagrelor) provide lower rates of ischemic events compared to clopidogrel, significant concerns remain about their onset of action. Moreover, their administration does not counterbalance the high residual platelet reactivity (HRPR) up to 4–6 h after the standard loading dose [20,21,22]. For this reason, strategies have been tested to increase the bioavailability of oral P2Y_12_ inhibitors, such as crushing or chewing tablets. However, pharmacokinetic and pharmacodynamic data remain limited [23,24,25]. So far, clopidogrel remains the P2Y_12_ inhibitor recommended in stable coronary artery disease (CAD) patients, unless specific high-risk procedural characteristics are present, such as complex left main or multivessel stenting, suboptimal stent deployment, or other conditions associated with high risk of stent thrombosis; in such cases, initial treatment with either prasugrel or ticagrelor may be considered according to European guidelines [26] if the tradeoff between risk of ischemia and bleeding is favorable [27]. All these therapies are limited by their need to be absorbed in the gastrointestinal (GI) tract before becoming available and this leads to an inevitable delay between drug intake and time of reaching effective platelet inhibition. Gastric emptying, intestinal motility, blood perfusion of the mucosa and its permeability are all factors influencing the absorption rate of medications [28]. Moreover, it has been reported that the velocity of platelet inhibition after oral intake was influenced by the clinical presentation: faster for stable CAD undergoing PCI, slower for NSTE-ACS patients, and the slowest for STEMI patients [20,29]. This phenomenon can be explained by a decreased cardiac output in ACS patients, which leads to a sympathetic system activation, and a vasoconstriction of the peripheral arteries that shunts the blood to vital organs, impairing gastric emptying, intestinal motility, and permeability of the hypo-perfused mucosa [30]. Elevated central pressure due to reduced cardiac output also leads to the release of atrial natriuretic peptide, which inhibits intestinal permeability and motility [31]. In acute settings, nausea and vomiting are common, reducing drug absorption as well. Finally, concomitant treatment with morphine, an opioid analgesic usually used to alleviate chest pain, delays gastric emptying, reduces intestinal peristalsis, and itself induces nausea and vomiting. Another barrier concerns the inability for oral administration of medications in intubated or unconscious patients. A new formulation of ticagrelor in orodispersible tablets that promptly releases its components upon contact with the oral cavity has recently become available and has been tested in a prospective trial of high-risk ACS patients. Although a superior grade of platelet inhibition was not obtained as compared with standard ticagrelor tablets, the trial confirmed the feasibility and safety of administration of ticagrelor without the need of swallowing water, that may prove to be convenient in critical ACS patients [32]. 

That said, following intake of oral P2Y_12_ inhibitors there is a variable timeframe of hours of inadequate antiplatelet protection. While the risk for ST is low with new generation stents, the delayed antiplatelet effects may still increase the risk of peri-procedural MI and impaired coronary/myocardial reperfusion, translating into worse clinical outcomes. Pre-treatment whenever possible could reduce this delay, but most recent ESC guidelines do not recommend (class III) pre-treatment with oral P2Y_12_ inhibitors in NSTE-ACS patients, because several trials showed no ischemic benefits and more bleeding complications [18]. In addition, treatment of stable CAD patients does not include a P2Y_12_ inhibitor before coronary angiography. These observations underscore the need to define strategies that can bridge the gap in platelet inhibitory effects following intake of oral P2Y_12_ inhibitors.

### 2.3. Parenteral P2Y_12_ Inhibitors

Parenteral administration of a P2Y_12_ inhibitor allows for immediate antiplatelet effects, skipping the delay and variability in intestinal absorption velocity and providing an enhanced platelet inhibition during the time window of inadequate response to oral agents. This is notable especially in high-risk patients undergoing PCI, who require an immediate platelet inhibition. 

Cangrelor is an adenosine triphosphate-analog that is a highly specific and a direct reversible antagonist for the P2Y_12_ receptor on the surface of platelets. This leads to blockage of ADP-induced GP IIb/IIIa receptors and inhibition of platelet aggregation. After administration, cangrelor does not need bioactivation and is immediately ready for platelet inhibition. It is available as a lyophilized powder and it is administered initially as a 30 mcg/kg intravenous bolus prior to PCI and then continued with a 4 mcg/kg/min infusion for at least 2 h or for the duration of PCI, whichever is longer. It reaches an immediate (~2 min) onset of action and has a very short offset with a rapid (30–60 min) restoration of platelet function after its discontinuation. There is neither dosage adjustment required for renal or hepatic impairment, nor for age. It has a short plasma half-life of 3–5 min as it is rapidly inactivated via dephosphorylation by nucleotidases in the blood and the major metabolite is considered inactive. Cangrelor allows high levels of platelet inhibition (>95%) and provides further decrease in platelet aggregation in patients treated than with the more potent oral P2Y_12_ inhibitors [33]. This reduces the risk of periprocedural and early postprocedural complications such as MI, repeat coronary revascularization and ST. Cangrelor is the only parenteral P2Y_12_ receptor inhibitor that has received approval. In 2015, both the US FDA and the EMA approved it in P2Y_12_ naïve patients undergoing PCI, both with ACS and with CAD. A large RCT showed faster and enhanced platelet inhibition in the peri-PCI period, translating into reduced ischemic events leading to clinical approval of the drug [34]. We will discuss later the CHAMPION program and more recent randomized clinical trials that have been designed to compare cangrelor vs. the more potent P2Y_12_ inhibitors (prasugrel and ticagrelor). 

Some parenteral antithrombotic drugs that interact with multiple pathways are currently being developed for the treatment of ACS, with the aim of further reducing ischemic events without significantly increasing bleeding complications [35]. Selatogrel is a reversible binding P2Y_12_ inhibitor formulated for subcutaneous (SC) administration. Its molecular structure derives from incorporation of the pyrimidine group of ticagrelor into a family of compounds previously studied as P2Y_12_ receptor antagonists [36,37]. Preclinical studies have suggested that selatogrel is potent and selective, but also that it may have a broader therapeutic index than clopidogrel or ticagrelor with regards to increased bleeding risk while maintaining antithrombotic effect [38]. Selatogrel has a rapid onset and one study of the radiolabeled drug suggested that there were no significant plasma metabolites, and that elimination was largely fecal, predicting no significant drug–drug interactions [39]. Phase II trials in both ACS and stable, chronic CAD are now being reported with promising results. Selatogrel reliably and potently inhibits platelet reactivity within 30 min after subcutaneous administration and for approximately 8 h in patients with chronic coronary syndrome, the effect fading within 24 h [40]. In patients with AMI, a single subcutaneous injection of selatogrel rapidly induced a profound and dose-dependent inhibition of platelet activity, independently from age, sex or clinical presentation, without major bleeding events and with short-term dyspnea as the only relevant adverse event [41]. The clinical context in which selatogrel may find its place remains to be determined; however, as it provides potent, rapid and reversible P2Y_12_ inhibition without the need for intravenous access or infusion, it could represent a promising pre-treatment option for early prehospital administration by healthcare professionals or even from self-administration by patients during a suspected re-infarction [42]. A large-scale clinical outcomes trial (SOS-AMI, Selatogrel Outcome Study in Suspected Acute Myocardial Infarction) in patients with a recent history of AMI, employing an autoinjector for early and convenient subcutaneous self-administration of selatogrel by the patient him/herself, is now ongoing (ClinicalTrials.gov Identifier: NCT04957719). 

RUC-4 (zalunfiban) is a second-generation GP IIb/IIIa inhibitor (GPI) which has shown a good safety profile and a high and limited-duration antiplatelet efficacy in both stable [43] and STEMI [44] patients. Zalunfiban is now being investigated in a large-scale Phase 3 RCT testing pre-hospital subcutaneous injection in STEMI patients (CELEBRATE, A Phase 3 Study of Zalunfiban in Subjects with ST-elevation MI, ClinicalTrials.gov Identifier: NCT04825743). 

## 3. Efficacy and Safety of Cangrelor: Main Evidence Available

### 3.1. The CHAMPION Program

The pharmacologic profile of cangrelor makes it not only an attractive agent for protection of ischemic events in patients undergoing PCI, but also a safe one in case of procedural complications, such as bleeding or need for emergent surgery, given its fast offset of effects, obviating the need for an antidote for reversal [45,46,47]. The efficacy and safety of cangrelor in the setting of PCI were evaluated in three large randomized controlled, double-blind, phase III trials (Table 1): 

The CHAMPION-PLATFORM trial enrolled 5362 patients with stable angina, unstable angina or NSTE-ACS undergoing PCI [48]. Patients were randomized to either cangrelor or placebo, bolus and infusion initiated during PCI, followed by 600 mg of clopidogrel at the end of the cangrelor infusion or at the end of the PCI for the placebo group. The primary endpoint of a composite of death, MI or ischemia-driven revascularization at 48 h was not significatively different between cangrelor or placebo (7.0 vs. 8.0%; *p* = 0.17) but cangrelor, had significantly lower rate of ST (0.2 vs. 0.6%; *p* = 0.02) and death from any cause (0.2 vs. 0.7%; *p* = 0.02) at 48 h. Cangrelor had no differences compared to placebo for major or minor bleeding according to the TIMI criteria and for severe or moderate bleeding according to the GUSTO study [49]. There was only a difference in major bleeding according to the ACUITY criteria, due to an excess of groin hematomas in the cangrelor group. However, the rates of blood transfusion were not significantly different. The CHAMPION-PCI trial (n = 8877) had a similar design to the prior trial but clopidogrel was given at the start of the placebo infusion, before PCI. The trial population was basically the same but also included ST-segment elevation myocardial infarction (STEMI) patients undergoing primary PCI (pPCI). The primary and secondary endpoints were the same as for CHAMPION-PLATFORM. However, in CHAMPION-PCI there were no statistically significant differences between the cangrelor and clopidogrel groups for any endpoint. The incidence of bleeding was significatively higher in the cangrelor group only by ACUITY minor (17.6 vs. 15.2%; *p* = 0.003) or GUSTO mild (19.6 vs. 16.9%; *p* = 0.001) criteria [50]. These discouraging results could be explained by the MI definition used in these trials which was considered obsolete and did not appropriately discriminate periprocedural MI especially from the first MI in ACS patients, being based mainly on CK and CKMB assays [51]. An analysis of these two trials using the universal MI definition demonstrated that the primary endpoint of a composite of death, MI and ischemia-driven revascularization was significantly reduced with cangrelor compared with the control (3.1 vs. 3.8%; *p* = 0.037). This difference was seen early, within the prior 6 h, according to the cangrelor time of action. Even acute ST was lower with cangrelor compared to placebo (0.2 vs. 0.4%; *p* = 0.018). In addition, cangrelor caused more rate of major and minor bleeding by ACUITY criteria and more hematomas, though they did not need more blood transfusions, according to trial results [52]. The benefit of cangrelor in ischemic endpoints seen in this analysis led to conduct of a similar trial, incorporating the universal definition of MI, the CHAMPION PHOENIX [34]. Enrolled patients (n=11,145), who were P2Y_12_ inhibitor naïve, underwent PCI for stable angina, NSTEMI or STEMI. They received cangrelor and a loading dose of clopidogrel (600 mg) at the end of infusion, or placebo and a loading dose of clopidogrel (300 or 600 mg) before or after PCI. Cangrelor led to a significantly lower rate of the primary endpoint (composite of death, MI, ischemia-driven revascularization or ST at 48 h) (4.7 vs. 5.9%; *p* = 0.005), particularly driven by a reduction in the periprocedural MIs; it led also to a significantly lower rate of the secondary endpoint of intraprocedural ST at 48 h [53]. Bleeding outcomes defined as GUSTO major and moderate criteria were not significantly different between the cangrelor group and the clopidogrel group. Bleeding measured using the more sensitive ACUITY criteria was consistently increased with cangrelor relative to clopidogrel in both stable and ACS patients. However, the need for blood transfusions was similar between the groups [53]. A post-hoc analysis combined the primary efficacy and safety endpoints to provide a composite of net adverse clinical events. Cangrelor compared with clopidogrel consistently reduced net adverse clinical events, in both ST and ACS subsets, both early at 48 h and at 30 days. These results were confirmed, at 48 h and 30 days, by a pooled analysis of all three CHAMPION trials [54].

### 3.2. Use of Cangrelor in Combination with Potent Oral P2Y_12_ Inhibitors

As already mentioned, the first trials of cangrelor mainly involved patients with stable or unstable CAD and a limited proportion of patients with STEMI. Thus, there was an urgent need for clinical and pharmacodynamic information on the wide use of cangrelor in combination with ticagrelor, the fastest oral formulation of P2Y_12_ inhibitors, for patients who have STEMI treated with pPCI. The CANTIC Study (Platelet Inhibition with Cangrelor and Crushed TICagrelor in STEMI Patients Undergoing Primary Percutaneous Coronary Intervention) was the first prospective randomized study designed in patients undergoing pPCI to explore the occurrence of drug–drug interaction (DDI) when cangrelor or placebo are concomitantly administered with ticagrelor [55]. Fifty STEMI patients scheduled for pPCI were randomized into two groups: one group received blinded 2 h cangrelor bolus followed by infusion, the other group received placebo. Additionally, both groups received 180 mg of crushed ticagrelor. Platelet reactivity was measured with VerifyNow P2Y_12_ point-of-care testing as P2Y_12_ reaction units (PRU) and vasodilator-stimulated phosphoprotein (VASP). Following PCI, all patients were prescribed aspirin indefinitely and ticagrelor 90 mg twice daily for at least 12 months. PRU levels were significantly lower in patients randomized to cangrelor than in those randomized to placebo as early as 5 min after the bolus (*p* < 0.001). PRU levels at 30 min (primary endpoint) were significantly lower with cangrelor versus placebo (63 vs. 214; *p* < 0.001) and remained significantly lower in the cangrelor group until completion of the 2 h infusion. In the placebo group, PRU levels decreased over time, with significant differences from baseline observed only 1 h after drug administration (*p* < 0.001), which became more marked after 2 h (*p* < 0.001). At the end of the infusion, there was an increase in PRU levels in the cangrelor group with significant differences at 1 h (*p* = 0.001) and 2 h (*p* = 0.027) after the infusion. In the placebo group, PRU levels continued to decrease at 1 h (*p* = 0.059) and 2 h (*p* = 0.007) after the infusion and remained similar at 1 and 2 h after having stopped the infusion. Rates of high platelet reactivity (HPR), as defined by PRU > 208, were significantly higher with placebo than with cangrelor at any study time point during the infusions (Table 2). A DDI during concomitant administration of cangrelor and ticagrelor was therefore ruled out, since no differences in PRU levels were found between the two groups after drug infusion was stopped. Indeed, patients in the cangrelor group did not have HPR, differently than placebo group where HPR status was reduced but still present in already half of the individuals at the end of the PCI and in one-third of the patients at the end of the placebo infusion. HPR levels were low overall and similar between groups after discontinuation of drug infusion. This consideration is consistent with the absence of DDI between cangrelor and ticagrelor. Despite several limitations, including the limited number of patients, the results were consistent with another nonrandomized pharmacodynamic study of the combination of cangrelor and ticagrelor for pPCI [56] and with a smaller open-label randomized trial [57]. The study demonstrated that in patients undergoing pPCI, the combination of cangrelor and ticagrelor results in a more rapid and potent platelet inhibitory effect compared to ticagrelor alone, with important implications for clinical practice such as a more versatile use of ticagrelor with respect to timing of its administration in patients treated with cangrelor.

The findings of the CANTIC study were confirmed by the recently published results of the prospective, randomized, double-blind, placebo-controlled, crossover, pharmacokinetic (PK) and pharmacodynamic (PD) SWAP-5 (Pharmacodynamic and Pharmacokinetic Profiles of Switching Between Cangrelor and Ticagrelor Following Ticagrelor Pre-treatment: The Switching Antiplatelet-5 Study) trial, which aimed to rule out DDI among cangrelor-treated patients who were pre-treated with ticagrelor [58]. Indeed, many patients in real-world clinical practice, in whom there may be the desire to use cangrelor to achieve enhanced P2Y_12_ inhibitory effects during PCI, are pre-treated with ticagrelor [59]. This may include patients in whom the full antiplatelet effects of ticagrelor may be delayed by several hours due to impaired absorption such as in patients presenting with ACS, especially STEMI, or treated with opioids [60,61]. In ticagrelor-pretreated patients there was a significant reduction in PRU at 30 min and 1 h after the start of the cangrelor infusion compared to the placebo group. At 2 h after stopping the cangrelor or placebo infusion, PRUs were low and similar in both groups (16.9 vs. 12.6), satisfying the primary endpoint of non-inferiority. No differences were found in PK/PD profiles such as plasma levels of ticagrelor and its metabolite between the two groups after drug infusion discontinuation, thus the absence of a DDI was also confirmed [58]. SWAP-5 Study was conducted in patients with stable CAD and not in patients with ACS undergoing PCI. Hence, the magnitude of the PK/PD findings observed may not be reflective of those in the acute setting. Several other studies are ongoing and will provide further insights into the use of cangrelor in patients undergoing pPCI. More data on transition to potent oral P2Y_12_ receptor inhibitors is desirable, for instance for patients who require a fast-acting intravenous agent such as cangrelor in emergency situations, such as cardiac arrest or cardiogenic shock, or for those who have been preloaded with oral antiplatelet agents and have angiographic findings requiring an additional antiplatelet agent. 

The first results of the CAMEO Registry, aimed at retrospectively addressing optimal platelet inhibition during early management of patients with MI prior to coronary angiography or coronary artery bypass grafting, demonstrated inter-hospital variability in how cangrelor was administered and switched to an oral P2Y_12_ inhibitor [62]. These findings highlight opportunities for optimization of cangrelor dosing, infusion duration, and the transition of care from the catheterization lab to the coronary intensive care unit. Data from recently published Cangrelor OHCA (Out-of-Hospital Cardiac Arrest) Study showed that in comatose survivors of OHCA undergoing PCI and target temperature management, cangrelor safely induced immediate and profound platelet inhibition without significant DDI with ticagrelor; nevertheless the study is a single-center and non-placebo-controlled trial [63]. Furthermore, the ongoing multicenter, randomized, double blind trial DAPT-SHOCK-AMI (Dual Antiplatelet Therapy for Shock Patients with Acute Myocardial Infarction; ClinicalTrials.gov Identifier: NCT03551964) will provide results on the comparison between the combination of cangrelor and crushed ticagrelor versus ticagrelor alone in patients with AMI complicated by initial cardiogenic shock and treated with pPCI. The ARCANGELO (Italian Prospective Study on Cangrelor) is a recently published multicenter, observational, prospective cohort study that included patients with ACS undergoing PCI who had not received an oral P2Y_12_ inhibitor before the PCI procedure and in whom oral therapy with P2Y_12_ inhibitors was not feasible or desirable; this study aimed to assess the safety of cangrelor in daily practice [64]. The primary endpoint is the incidence of any hemorrhage, according to Bleeding Academic Research Consortium (BARC) criteria, in the 30 days following the PCI, calculated as the ratio between the number of patients experiencing at least one event during the 30-day observation period and the total number of evaluable patients. The different types of bleedings according to the GUSTO criteria and MACE at various timeframes (from 48 h to 30 days) were investigated, too. The preliminary results showed that all bleedings were classified as BARC Type 1–2, BARC Grade 3a bleeding occurred in one (0.3%) patient, while more severe bleedings were not reported. A total of 17 bleedings were observed in the 320 patients who completed the study. MACE was observed in four patients (two AMI, one sudden cardiac death, one non-cardiovascular death). None bleeding was classified as related to cangrelor. The final analysis of data will assess a more precise evaluation of the study endpoints; however, the use of cangrelor in patients with ACS undergoing PCI does not appear to be associated with severe bleedings. The ongoing SWAP-6 (Pharmacodynamic and Pharmacokinetic Profiles on Switching from Cangrelor to Prasugrel in Patients with Acute Coronary Syndrome Undergoing Percutaneous Coronary Intervention: The Switching Antiplatelet-6 Study; ClinicalTrials.gov Identifier: NCT04668144) trial will further clarify pharmacodynamic effects to rule out a DDI when cangrelor and prasugrel are concomitantly administered in patients undergoing coronary stenting. Currently, a single study has suggested that prasugrel can be administered at the beginning of the cangrelor infusion, with no evidence of drug interactions [65]. Whether this evidence applies to patients with STEMI is unknown and this treatment strategy remains off-label [66].

FABOLUS-FASTER (Facilitation through Aggrastat or Cangrelor Bolus and Infusion over Prasugrel: A Multicenter Randomized Open-Label Trial in Patients with ST-Elevation Myocardial Infarction Referred for Primary Percutaneous Intervention) is a trial that compared, for the first time, the pharmacodynamic effects of cangrelor with the GPI inhibitor tirofiban and the pharmacodynamic and pharmacokinetic effects of prasugrel 60 mg in chewed or whole tablets in patients with STEMI undergoing pPCI [67]. Patients were randomly assigned (1:1:1) to cangrelor (*n* = 40), tirofiban (*n* = 40) (both given as a bolus and 2 h infusion followed by a loading dose of 60 mg prasugrel at the time of infusion interruption) or prasugrel 60 mg loading dose (*n* = 42). Patients in the prasugrel group underwent further 1:1 sub-randomization to oral administration of the loading dose as chewed (*n* = 21) or whole (*n* = 21) tablets. Briefly, the aim of the study was to test three primary hypotheses: non-inferiority of cangrelor versus tirofiban, superiority of both tirofiban and cangrelor versus chewed prasugrel, and superiority of chewed prasugrel versus whole prasugrel. Cangrelor did not reach non-inferiority as compared to tirofiban in terms of ADP-induced platelet aggregation (Table 3) due to a lower platelet aggregation in patients treated with tirofiban than cangrelor or chewed prasugrel up to 2 h. Interestingly, residual platelet reactivity was lower with cangrelor compared to chewed prasugrel within the first hour, but higher thereafter.

Tirofiban was associated with lower TRAP-induced platelet aggregation than cangrelor or chewed prasugrel (*p* < 0.001 at any time point for both comparisons) whereas there was no difference between cangrelor and chewed prasugrel or between the two prasugrel groups. The FABOLUS-FASTER study strengthened the notion of the superiority of parenteral over oral antiplatelet drugs in the acute phase of STEMI treatment in terms of platelet inhibition; however, the observed superiority of tirofiban versus cangrelor remains a mechanistic observation, and whether it could be translated into better clinical outcomes without impairing risk of bleeding remains to be elucidated. Large-scale studies re-evaluating the comparative risks and benefits of a short infusion of parenteral platelet inhibitors such as cangrelor or GPI versus the newer oral P2Y_12_ receptor blockers alone in contemporary pPCI practice remain desirable. Based on the observations from the FABOLUS-FASTER that cangrelor followed by prasugrel is associated with a rebound in platelet activation over 2 to 4 h and on the data of CANTIC Study [55] and Alexopoulos [20] showing some HRPR during and after the cangrelor infusion, it could be hypothesized that when cangrelor is used, ticagrelor may be the preferred oral P2Y_12_ inhibitor. 

## 4. Current Recommendations for the Transition from Cangrelor to Oral P2Y_12_ Inhibitors

At the end of cangrelor infusion, which should be prolonged at least for two hours, patients who underwent PCI with stent implantation should receive a loading dose of an oral P2Y_12_ inhibitor, beyond aspirin. The timing for the P2Y_12_ inhibitor loading dose is related to the pharmacology of the specific drug. Clopidogrel active metabolite is rapidly degraded if it does not bind P2Y_12_ receptor. So, if the receptor is already occupied by cangrelor, a more potent P2Y_12_ inhibitor, clopidogrel active metabolite is degraded, getting no platelet inhibition following the cangrelor infusion cessation. Therefore, loading dose of 600 mg clopidogrel must be administered only after cangrelor infusion cessation. This is also widely supported by CHAMPION platelet sub-study, where there was no apparent significant pharmacodynamic interaction when clopidogrel was administered at the end of the cangrelor infusion [68].

Prasugrel is a thienopyridine requiring activation with similar pharmacodynamics to clopidogrel. Therefore, the loading dose administration of prasugrel should be administered at the end of cangrelor infusion as well. A study examining the transition from cangrelor to thienopyridines showed a transient recovery of platelet reactivity during the switch and found the optimal administration time of prasugrel, to limit the recovery of platelet function, at 30 min prior to cangrelor cessation [69]. This is in line with the more potent binding power to P2Y_12_ receptor of prasugrel compared to clopidogrel. In accordance with this study, the EMA recommends the administration of a prasugrel loading dose (60 mg) either 30 min prior to the cangrelor cessation or immediately after; the FDA recommends it only immediately after cangrelor cessation. 

Ticagrelor is a reversible P2Y_12_ inhibitor, and it binds a different site of the receptor compared to cangrelor. Previous studies have demonstrated that there are no DDI between ticagrelor and cangrelor, suggesting that ticagrelor can be given at any time during cangrelor infusions or at the end of it [70]. Both the FDA and EMA have recommended the administration of a ticagrelor loading dose (180 mg) either during the cangrelor infusion or immediately after the infusion cessation. 

For clopidogrel and prasugrel, the recommended transitions from cangrelor may result in a brief inadequate P2Y_12_ inhibition, due to the delayed onset of action of clopidogrel and prasugrel. This is consistent with the results of a recent observational pharmacodynamic registry confirming that the switch from cangrelor to clopidogrel could expose patients to a variable period of inadequate platelet inhibition, while ticagrelor given as early as possible after starting cangrelor infusion may avoid any rebound effect in platelet reactivity [71]. Therefore, it is reasonable to prefer ticagrelor as the maintenance P2Y_12_ inhibitor in oral DAPT, as it can be started prior to the cessation of cangrelor.

## 5. Antiplatelet Bridging for CABG and Non-Cardiac Surgery

**Figure 1:**
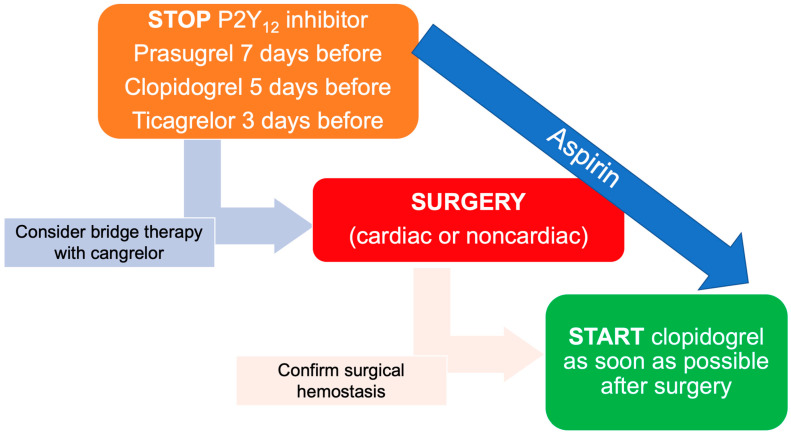
Time frames of P2Y_12_ inhibitor discontinuation and restarting in patients undergoing cardiac or non-cardiac surgery. Elaborated from Valgimigli et al. [72].

Patients treated with a P2Y_12_ inhibitor, who require a major cardiac or non-cardiac surgery, have worse outcomes due to an increased risk for peri- and post-operative bleedings, reoperation and need for blood transfusions. The European guidelines recommended to delay, if it is possible, a non-emergent surgery after PCI with DES implantation until completion of the full course of DAPT, or at least after one month of DAPT [72]. In cases when surgery cannot be delayed for a longer period, a minimum of 1 month of DAPT should be considered, because the higher risk of adverse cardiac events is within the first 30 days after PCI. In any case of patients who need earlier surgery, it is recommended to withhold P2Y_12_ inhibitor at least 7 days for prasugrel, 5 days for clopidogrel and 3 days for ticagrelor before surgery (Figure 1). However, cessation of DAPT in the setting of recent ACS or PCI with stent implantation is associated with a time-dependent increased risk for worse outcomes. It is particularly true for ACS patients with high ischemic risk features, who need at least 6 months of DAPT. 

For patients with a very high risk of ST who cannot delay surgery, bridging therapy with intravenous, reversible platelet inhibitor may be considered (Figure 1). Due to its profile of being a rapid onset/offset, potent and reversible P2Y_12_ inhibitor, cangrelor was tested as a P2Y_12_ inhibitor ‘bridge’ after discontinuation of thienopyridines, in patients undergoing surgery (BRIDGE trial). In this trial, 210 participants who were planned to undergo non-emergent CABG and had received an oral P2Y_12_ inhibitor were randomized to either cangrelor as bridge therapy or placebo. Aspirin therapy was maintained. Cangrelor, compared with placebo, resulted in higher proportions of suppressed platelet activity, without a significant increase in CABG-related bleeding (11.8 vs. 10.4%; *p* = 0.76), despite some participants receiving cangrelor infusion for up to 7 days [73].

Based on the BRIDGE trial protocol, a recent consensus document standardized management of antithrombotic therapy in patients treated with coronary stents in various types of surgery [74]. It is recommended to stop prasugrel 7 days, clopidogrel and ticagrelor 5 days before surgery. Cangrelor as bridge therapy should be started within 72 h from P2Y_12_ discontinuation, at the dose of 0.75 μg/kg/min without bolus and continued until 1–6 h before skin incision. Clopidogrel should be started, with a new loading dose of 300 or 600 mg, as soon as possible after surgery (within 1–6 h). If oral administration is not possible due to intubation, cangrelor should be restarted. Prasugrel and ticagrelor are discouraged. The MONET BRIDGE study was designed to assess the use of cangrelor as a platelet-inhibiting bridge for patients who discontinue DAPT before cardiac and non-cardiac surgery within 12 months from coronary stent implantation [75]. It demonstrated that perioperative bridging therapy with cangrelor is a feasible approach for patients with DES at high thrombotic risk and undergoing surgery requiring interruption of DAPT: no ischemic outcomes occurred after surgery and up to 30-days follow-up. Moreover, the mean hemoglobin drop was <2 g/dL; nine patients received blood transfusions consistent with the type of surgery, but no life-threatening or fatal bleeding occurred. More studies are warranted to support the efficacy and safety of a standardized bridging strategy by identifying the patient population that would receive the maximum clinical benefit from bridge therapy. In addition to MONET BRIDGE, the MARS (Management of Antiplatelet Regimen During Surgical Procedures; ClinicalTrials.gov Identifier: NCT03981835) trial is currently studying the area of perioperative antiplatelet therapy management through a multi-center, observational US national registry designed to collect preoperative, intraoperative and postoperative clinical strategies, therapeutic interventions, and 30-day outcomes data of ~1500 patients post-PCI scheduled to undergo cardiac or noncardiac surgery.

## 6. Future Directions

The current available oral P2Y_12_ has a relatively slow onset of action, so drug-naïve patients, and especially those with ACS, undergoing PCI lack the protection conferred by antiplatelet therapy for a too long period and may be exposed to a greater thrombotic risk. Cangrelor proved its effectiveness in drug-naïve patients undergoing PCI, both in the stable and the acute setting, by reducing early and 30-day ischemic outcomes, with particular emphasis for ischemia driven revascularization and early ST. Cangrelor appears to be a very safe drug with a low rate of bleeding and specifically of major (BARC 3–5) events. The results of ongoing randomized trials with new short-acting and potent parenteral antiplatelet agents will be likely to open a new debate about the optimal choice and timing to administer parenteral DAPT in patients with STEMI, since we could have available, at the same time, a drug to be self-administered at home (selatogrel), a drug to be administered in the ambulance (zalunfiban) and a drug to be administered in the hospital (cangrelor). Further RCTs are needed about the combination of parenteral and potent oral P2Y_12_ inhibitors in patients with ACS and about the optimal switching strategies. Available studies so far support the most adopted practice to administer ticagrelor at the same time of cangrelor bolus or as soon as possible after initiation of cangrelor infusion. 

## Figures and Tables

**Table 1:** Overview of the CHAMPION Program trials.

	CHAMPION PLATFORM	CHAMPION PCI	CHAMPION PHOENIX
Years	2007–2009	2007–2009	2010–2012
Patients (*n*)	5362	8877	11,145
Diagnosis	NSTE-ACS (94.8%); stable angina (5.2%)	STEMI (11.2%); NSTE-ACS (73.8%); stable angina (1.5%)	STEMI (18%); NSTE-ACS (25.7%); stable angina (62.3%)
Antiplatelet therapy	Clopidogrel naïve	Clopidogrel	Clopidogrel naïve
Treatment	Cangrelor: 30 μg/kg bolus, 4 μg/kg/min infusion	Cangrelor: 30 μg/kg bolus, 4 μg/kg/min infusion	Cangrelor: 30 μg/kg bolus, 4 μg/kg/min infusion
Transition to clopidogrel	Clopidogrel 600 mg at the end of cangrelor infusion	Clopidogrel 600 mg at the end of cangrelor infusion	Clopidogrel 600 mg at the end of cangrelor infusion
Control arm	Placebo	Clopidogrel 600 mg	Clopidogrel 600 mg or 300 mg
Definition of myocardial infarction	Clinical	Clinical	Universal definition
Primary composite endpoint	Death, MI, IDR at 48 h	Death, MI, IDR at 48 h	Death, MI, IDR at 48 h
Results	OR 0.87 (95% CI 0.71–1.07; *p* = 0.17)	OR 1.05 (95% CI 0.88–1.24; *p* = 0.59)	OR 0.78 (95% CI 0.66–0.93; *p* = 0.005)

**Table 2:** Characteristics of patients randomized in the CANTIC trial [55].

	Cangrelor Group	Placebo Group	*p*-Value
Patients, n	22	22	
Diagnosis	STEMI	STEMI	
Treatment	Cangrelor: 30 μg/kg 2 h bolus, 4 μg/kg/min infusion	Placebo	
Time from bolus to end of PCI, min (SD)	39 (18–51)	33 (26–60)	
Transition to ticagrelor	Crushed ticagrelor 180 mg	Crushed ticagrelor 180 mg	
HPR at baseline, n HPR during cangrelor 5 min, n (%) 30 min, n (%) End of PCI, n (%) 1 h, n (%) 2 h, n (%) HPR post cangrelor 1 h, n (%) 2 h, n (%)	15 (68%) 0 (0%) 0 (0%) 0 (0%) 0 (0%) 0 (0%) 2 (10%) 1 (5%)	15 (68%) 15 (71%) 12 (57%) 13 (62%) 8 (38%) 6 (33%) 2 (12%) 1 (6%)	NS <0.001 <0.001 <0.001 0.003 0.007 NS NS

**Table 3:** Rates of high residual platelet reactivity (>59%) at Light Transmittance Aggregometry (LTA) after ADP 20 μmol/L stimulation in FABOLUS FASTER trial [67].

	Rates				*p*-Values			
	Tirofiban	Cangrelor	Chewed Prasugrel	Integral Prasugrel	Tirofiban vs. Cangrelor	Tirofiban vs. Chewed Prasugrel	Cangrelor vs. Chewed Prasugrel	Chewed Prasugrel vs. Integral Prasugrel
>59% LTA with ADP 20 µmol/L 15 min 30 min 1 h 2 h 3 h 4 to 6 h	0.0% 0.0% 0.0% 0.0% 7.5% 7.5%	57.5% 55.0% 55.0% 50.0% 81.6% 68.4%	100.0% 90.5% 66.7% 38.1% 28.6% 33.3%	95.2% 95.2% 81.0% 52.4% 19.0% 19.0%	<0.001 <0.001 <0.001 <0.001 <0.001 <0.001	<0.001 <0.001 <0.001 <0.001 0.030 0.014	<0.001 0.012 NS NS <0.001 0.009	NS NS NS NS NS NS

## Data Availability

Not applicable.
